# Woody forages effect the intestinal bacteria diversity of golden pompano *Trachinotus ovatus*

**DOI:** 10.1186/s13568-018-0550-2

**Published:** 2018-02-27

**Authors:** Biao Chen, Liu-ling Gao, Qing Pan

**Affiliations:** College of Marine Sciences, South China Agriculture University, 483 Wu Shan Road, Guangzhou, 510642 Guangdong Province China

**Keywords:** Golden pompano, Woody forages, Intestinal bacteria, Illumina sequence

## Abstract

To understand the effect of woody forages on golden pompano (*Trachinotus ovatus*) intestinal bacteria diversity and exploit new aquafeed ingredients, the diets of *Moringa oleifera* Lam (MOL), *Broussonetia papyrifera* (BP), *Neolamarckia cadamba* (NC) and *Folium mori* (FM) formulated with 70% of reference (Ref) diet and 30% of the four woody plants leaves were fed to golden pompano with initial body weight of 34.4 ± 0.5 g for 56 days, respectively. Bacteria diversity of golden pompano intestine and tank water (W) samples were analyzed using high-throughput illumina sequencing and the result indicated that the dominate phyla of golden pompano intestine were *Proteobacteria*, *Bacteroidetes*, *Firmicutes* and *Fusobacteria*. *Proteobacteria* in BP was significantly higher than those in NC (*P* < 0.05). *Firmicutes* in NC were significantly higher than those in BP and FM (*P* < 0.05). At genera level, *Lactobacillus* in NC was significantly higher than those in BP, MOL and FM groups (*P* < 0.05). The PCoA and heat map analyses showed that the intestinal bacteria community of golden pompano fed with woody forages and Ref diet presented higher similarity and the bacteria community of golden pompano intestine were clearly distinguished from those of W. Phylogenetic investigation of communities by reconstruction of unobserved states showed that the intestinal bacteria dominant metabolism pathways of golden pompano fed with woody forages and Ref diet were biosynthesis of amino acids and carbon metabolism. Overall, the present study first successfully characterized the intestinal bacteria diversity of golden pompano.

**Electronic supplementary material:**

The online version of this article (10.1186/s13568-018-0550-2) contains supplementary material, which is available to authorized users.

## Introduction

Golden pompano (*Trachinotus ovatus*) is a marine water species and mainly distributed in tropical and temperate seas (Niu et al. 2013). It has become a popular cultured species because of its fast growth and high flesh quality. Studies showed that the vertebrate intestine inhabited diverse bacteria which had a mutual relationship with host and played key functions in nutrition (Hassaan et al. 2017) and development (Verner-Jeffreys et al. 2003). Meanwhile, studies also found that *Bacteroidetes*, *Firmicutes* and *Fusobacteria* in intestine could produce digestive enzymes (Becker et al. 2014). However, there is not report about the golden pompano intestinal bacteria diversity.

Aquaculture has become the fastest growing food-producing sector which contributed 50.9% to total global fisheries production (Hamdan et al. 2016). The increased production of intensively reared fish species necessitates the exploitation of new feedstuff resources (Adeoye et al. 2016). Some woody materials with high nutrition and low price may be the most promising new aquatic feed ingredients. *Moringa oleifera* Lam leaves have moderately high level of protein, amino acid, vitamin A, iron and calcium (Nahid et al. 2003). Meanwhile, studies indicated that the *Moringa oleifera* Lam could be used to substitute 10% of fishmeal in Nile tilapia diets without significant reduction on growth (Afuang et al. 2003). *Broussonetia papyrifera* leaves contain lots of biologically active compounds which were benefit to the immunity of organism (Xi et al. 2013). *Folium mori* leaves and *Neolamarckia cadamba* leaves have 16.8 and 20.9% crude protein (CP), respectively (Doi et al. 2000).These woody plants with high nutritional value may be ideal new diets sources in aquaculture. However, several studies indicated feedstuff could influence intestinal bacteria diversity, such as, dietary malic acid (Hassaan et al. 2017), thymus vulgaris essential oil (Navarrete et al. 2010) and soybean meal (Merrifield et al. 2009). These findings indicate that further studies on intestine bacteria dynamics of golden pompano fed with new source woody forages is needed.

In the present study, the intestine bacteria of golden pompano was first analyzed by illumina-based high-throughput sequencing, which would contribute to understand the intestinal bacteria diversity and dynamics of golden pompano fed with woody forages and be helpful to the exploitation of woody forges in aquaculture.

## Materials and methods

### Experimental design and sampling produces

The formulation and proximate composition of Ref diet were showed in Table 1 which contained 34.3% crude protein (CP) and 7.0% crude lipid (EE). Fishmeal and soybean meal were used as major protein sources. Menhaden fish oil, sunflower oil and soybean lecithin were used as lipid sources. The reference (Ref) diet was prepared according to the protocol of Pan et al. (2003). And the mixed wet mash was extruded into pellet diet with 3.5 mm in diameter by twin screw extruders (SLX-80, Guangzhou, China). The diets of *Moringa oleifera* Lam (MOL), *Broussonetia papyrifera* (BP), *Neolamarckia cadamba* (NC) and *Folium mori* (FM) were formulated with 70% of Ref diet and 30% leafmeals of *Moringa oleifera* Lam, *Broussonetia papyrifera*, *Neolamarckia cadamba* and *Folium mori*, respectively. The amino acid and nutrients composition of MOL, BP, NC and FM were shown in Table 2. And the nutrients composition of MOL, BP, NC and FM diets were showed in Table 3.

**Table 1:** Formulation and proximate composition of Ref diet

Formulation	%
Fishmeal	31.0
Soybean meal	21.0
Menhaden fish oil	2.8
Sunflower oil	1.0
Soybean lecithin (50%)	1.5
Wheat flour	40.2
Ca(H_2_PO_4_)_2_–H_2_O	1.5
Taurine	0.5
Choline chloride (50%)	0.4
Vitamin premix^a^	0.5
Mineral premix^b^	0.5
Y_2_O_3_/Cr_2_O_3_	0.1
Nutrients composition analyses	
Moisture	10.1
Crude protein	34.3
Crude lipid	7.0
Ash	7.0
Energy (KJ/g)	19.5

^a^Vitamin premix (g/kg) and ^b^ Mineral premix (g/kg) were supplied by Guangzhou Chengyi Aquatic Technology Company

**Table 2:** Analyzed amino acid and nutrients composition of MOL, FM, BP and NC

Items	MOL	FM	BP	NC
Aspartic acid (%)	2.4	1.7	2.0	1.2
Threonine	1.2	0.8	0.8	0.6
Serine	1.1	0.8	0.8	0.6
Glutamic acid (%)	3.2	1.9	1.9	1.6
Proline	1.2	0.9	1.0	0.6
Glycine	1.4	0.9	1.0	0.7
Alanine	1.7	1.1	1.1	0.7
Valine	1.4	1.0	1.0	0.7
Methionine (%)	0.1	0.1	0.1	0.1
Isoleucine (%)	1.2	0.8	0.8	0.6
Leucine	2.3	1.4	1.5	1.1
Tyrosine	0.8	0.5	0.5	0.4
Phenylalanine (%)	1.6	0.9	1.0	0.7
Lysine	1.6	1.1	1.1	0.7
Histidine	0.6	0.3	0.4	0.3
Arginine	1.5	0.9	1.0	0.7
Total amino acid (%)	23.1	15.1	15.8	11.1
Moisture (%)	1.9	0.9	1.4	1.5
CP (%)	26.6	17.9	18.3	13.4
EE (%)	4.3	4.4	3.5	2.7
Ash (%)	10.8	11.6	16.0	7.6
Energy (KJ/g)	17.8	16.9	15.4	18.7

**Table 3:** Analyzed nutrients composition of MOL, BP, NC and FM diets

Nutrients composition analysis	MOL	BP	NC	FM
Moisture (%)	10.6 ± 0.0	10.4 ± 0.1	11.0 ± 0.0	11.0 ± 0.1
Crude protein (%)	33.1 ± 0.1	30.7 ± 0.1	29.2 ± 0.1	31.0 ± 0.1
Crude lipid (%)	6.4 ± 0.0	7.3 ± 0.0	6.8 ± 0.0	6.4 ± 0.0
Ash (%)	8.3 ± 0.1	8.7 ± 0.1	6.9 ± 0.3	9.6 ± 0.0
Energy (KJ/g)	19.4 ± 0.0	19.2 ± 0.1	19.4 ± 0.2	19.0 ± 0.2

The experimental golden pompano were obtained from Shenzhen Base of South China Sea Fisheries Research Institute and fed with Ref diet and woody forages for 1 week to acclimatize experimental conditions and feed. Then 300 healthy fish with initial weight of 34.4 ± 0.5 g were randomly stocked in 15 tanks with 20 fishes in each aquariums (500 L water capacity). Each test diet was fed to fish in three parallel aquariums. All golden pompano were fed with diet at 07:30, 12:30 and 18:00 daily by hand to apparent satiation for 56 days and the woody forages did not influence the feeding in the present study. 1/4–1/3 of the water in aquariums was changed with the filterable sea water every day. During the feeding trial, water temperature ranged at 24–26 °C, pH at 7.6–7.8, salinity at 15–17 g/L, N–NH_4_^−^ < 0.1 mg/L and DO > 5 mg/L.

After the feeding trial, golden pompano were fasted 7 h. Three fish were randomly picked up from each aquarium and sacrificed with tricaine methanesulfonate (MS-222). Then the fish were dissected with sterile scissors and intestines were filled with chyme. Intestinal contents were carefully collected to 1.5 mL sterile centrifuge tube. In addition, 300 mL tank sea water (W) sample was collected and filtered by 0.22 μm pore size hydrophilic polyethersulfone membrane filter. All samples were stored at − 80 °C. In addition, the proximate composition analyses of five diets were based on the Official Analytical Chemists (AOAC 1995).

### DNA extraction and PCR amplification

The microbial DNA of golden pompano intestine and tank water was extracted using EZNA Stool DNA Kit (Omega Bio-tek). The V3–V4 region of bacteria 16S ribosomal RNA gene was amplified by PCR using the primers V338F (5′-ACTCCTACGGGAGGCAGCAG-3′) and V806R (5′-GGACTACHVGGGTWTCTAAT-3′). All PCR amplifications were performed in triplicate at 20 μL reactions containing 4 μL of 5× FastPfu buffer, 2 μL of 2.5 mM dNTPs, 0.8 μL of each primer (5 μM), 0.4 μL (1 unit) of FastPfu polymerase (TransGen AP221-02: TransStart™ FastPfu DNA polymerase, TransGen Biotech, Beijing, China) and 10 ng of template DNA. The thermal cycling program was performed as followings: 95 °C for 5 min, followed by 25 cycles at 95 °C for 60 s, 50 °C for 60 s, 72 °C for 60 s and a final extension at 72 °C for 7 min. The PCR products were examined using 1.8% agarose gel and excised and purified using the QIAquick Gel extraction kit (Qiagen, Hilden, Germany) according to the manufacture protocol. Purified amplicons were pooled in equimolar quantities and sequenced with Illumina HiSeq 2500 platform.

### Sequence analyses

Paired-end reads were merged with FLASH v1.2.7 according to overlap more than 10 bps. Raw Tags were filtered by Trimmomatic v0.33 and chimeric sequences were removed by UCHIME v4.2. Effective Tags were clustered at a 97% sequence identity into operational taxonomic units (OTUs) using UCLUST in QIIME (version 1.8.0) software package (Edgar 2010; Caporaso et al. 2010). Each OTU were aligned to SILVA bacteria database using PyNAST (Koetschan et al. 2014). Taxonomic OTU assignments were accomplished by Ribosomal Database Project (RDP) Classifier with a minimum confidence of 80% (Caporaso et al. 2010).

Heatmap was analyzed through “R vegan package” (Kang et al. 2013). Abundance-based coverage estimator (ACE), Chao 1, Shannon, Simpson and Good’s coverage indices were used to analyze the richness and diversity. Rarefaction curves were analyzed with MOTHUR (version v.1.30). Principal coordinate analyses (PCoA) were based on the binary Jaccard distances (Lozupone and Knight 2005). Gene prediction was used with the phylogenetic investigation of communities by reconstruction of unobserved states (PICRUSt) and Greengenes database v13.5 (de Oliveira et al. 2013; Parks et al. 2014).

### Statistical analyses

The results were analyzed by One-way ANOVA at 5% significance level using SPSS version 20.0 (SPSSInc, Chicago, IL) and shown as mean ± SE.

### Nucleotide sequence accession number

The raw reads were deposited to the NCBI Sequence Read Archive (SRA) database under accession number SRP115358.

## Results

### Statistical analysis of sequences

**Fig. 1:**
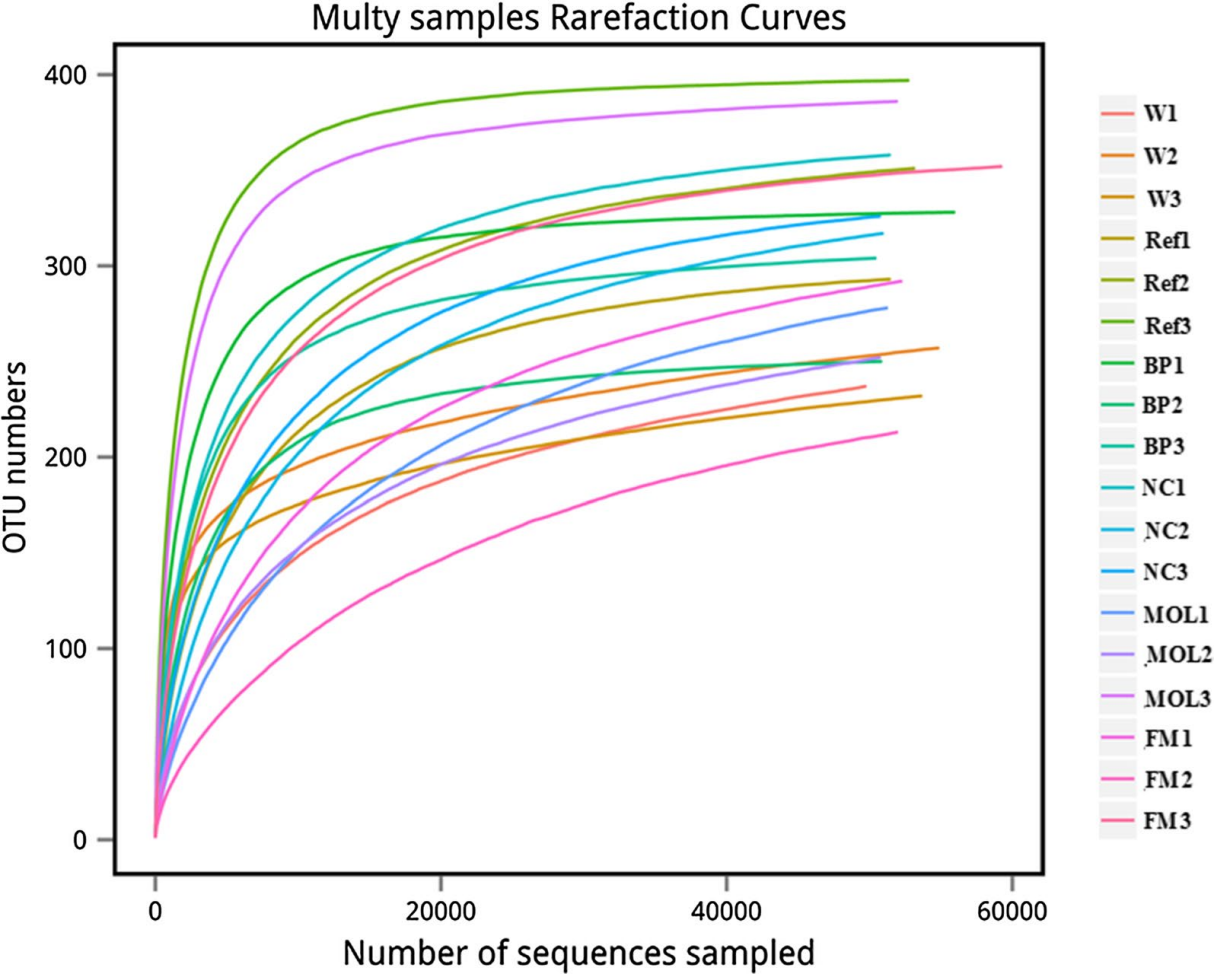
Rarefaction analyses of all samples. Rarefaction curves represented the number of operational taxonomic unit (OTU) detected in W (W1, W2, W3), Ref (Ref1, Ref2, Ref3), BP (BP1, BP2, BP3), NC (NC1, NC2, NC3), MOL (MOL1, MOL2, MOL3) and FM (FM1, FM2, FM3). Sequences were clustered at 97% sequence similarity

1,086,123 effective tags were obtained from 18 samples. The average lengths of effective tags were 422 bps. A total of 5092 OTUs at 97% sequence similarity were obtained, with average of 282 OTUs in each sample. Rarefaction curves indicated that the obtained sequence could reflect majority of bacteria diversity in each sample (Fig. 1).

Table 4 presented the alpha diversity of W, Ref, BP, NC, MOL and FM. The ACE and Chao 1 indices of W, Ref, BP, NC, MOL and FM ranged from 279.0 ± 29.1 to 340.0 ± 28.5, 280.0 ± 29.3 to 342.0 ± 29.3, respectively. The Simpson and Shannon indices of W, Ref, BP, NC, MOL and FM ranged from 0.1 ± 0.0 to 0.3 ± 0.1, 2.7 ± 0.3 to 3.7 ± 0.3, respectively. The bacteria diversity and richness had not significant difference in W, Ref, BP, NC, MOL and FM (*P* > 0.05).

**Table 4:** OTUs, ACE, Chao 1, Simpson, Shannon and Good’s coverage for 16s rRNA libraries of all samples

Sample ID	W	Ref	BP	NC	MOL	FM
OTUs	229.0 ± 7.7	333.0 ± 29.7	276.0 ± 29.5	319.0 ± 11.7	279.0 ± 44.3	259.0 ± 44.8
ACE	281.0 ± 8.0	340.0 ± 28.5	279.0 ± 29.1	334.0 ± 13.7	313.0 ± 29.6	294.0 ± 31.7
Chao1	289.0 ± 13.8	342.0 ± 29.3	280.0 ± 29.3	339.0 ± 6.3	328.0 ± 20.0	290.0 ± 34.4
Simpson	0.3 ± 0.2	0.2 ± 0.1	0.1 ± 0.0	0.3 ± 0.1	0.2 ± 0.0	0.1 ± 0.1
Shannon	2.7 ± 0.7	3.3 ± 0.5	3.6 ± 0.0	2.7 ± 0.3	3.2 ± 0.7	3.7 ± 0.3
Good’s coverage	1.0 ± 0.0	1.0 ± 0.0	1.0 ± 0.0	1.0 ± 0.0	1.0 ± 0.1	1.0 ± 0.0

In the same row, values with different letter superscripts mean significant differences (*P* < 0.05)

### Taxonomic composition

**Fig. 2:**
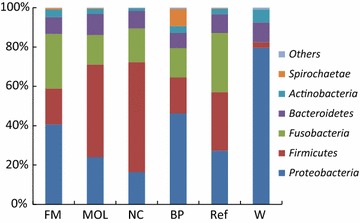
The intestinal bacteria communities at phyla level. The color-coded bar plot showed the percentages of intestinal bacteria communities in W, Ref, BP, NC, MOL and FM groups at phyla level. “Others” meant the relative abundance were less than 1%

A total of 15 phyla were detected in W, Ref, BP, NC, MOL and FM and the most abundant phyla were *Proteobacteria*, *Bacteroidetes*, *Firmicutes* and *Fusobacteria* (Fig. 2). The most abundant phyla in Ref were *Fusobacteria* (30.2%), *Firmicutes* (29.7%), *Proteobacteria* (27.3%) and *Bacteroidetes* (9.5%). *Proteobacteria* (39.1, 23.8, 16.4 and 46.1%), *Firmicutes* (17.5, 46.7, 55.9 and 18.2%), *Fusobacteria* (26.7, 14.8, 17.1 and 14.9%) and *Bacteroidetes* (8.2, 10.7, 9.0 and 7.7%) were the dominate phyla in FM, MOL, NC and BP, respectively. In W, the dominate phyla were *Proteobacteria* (79.6%), *Firmicutes* (2.8%), *Bacteroidetes* (10.1%). *Proteobacteria* in BP group was significantly higher than those in NC (*P* < 0.05). *Firmicutes* in NC were higher than those in BP and FM (*P* < 0.05). *Proteobacteria* in Ref, BP, NC, MOL and FM were significantly lower than those in W (*P* < 0.05). *Fusobacteria* in Ref, BP, NC, MOL and FM groups were significantly higher than those in W (*P* < 0.05).

**Fig. 3:**
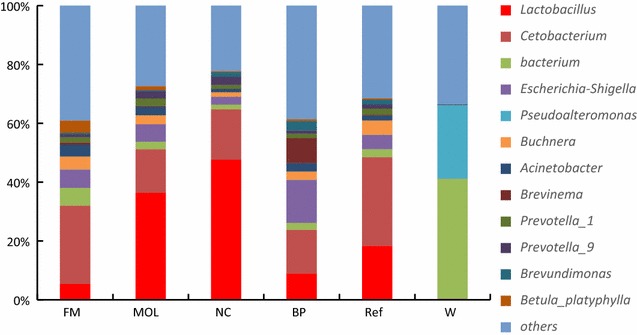
The intestinal bacteria communities at genera level. The color-coded bar plot showed the percentages of intestinal bacteria communities in W, Ref, BP, NC, MOL and FM groups at genera level. Others meant the relative abundance were less than 1%

At genera level, 224 bacteria genera were detected from all samples (Fig. 3). *Cetobacterium* (30.1%), *Lactobacillus* (18.4%), *Buchnera* (4.9%) and *Escherichia*–*Shigella* (4.9%) were the dominate genera in Ref group. *Cetobacterium* (26.6, 14.8, 17.1 and 14.8%), *Lactobacillus* (7.5, 20.8, 47.7 and 3.3%), *Buchnera* (4.3, 5.9, 2.6 and 14.6%) and *Escherichia*–*Shigella* (6.3, 5.9, 2.6 and 14.6%) were the dominate genera in FM, MOL, NC and BP groups. In W, *Pseudoalteromonas* (0.6%) and *Lactobacillus* (0.5%) were the dominate genera. *Lactobacillus* in NC were significantly higher than those in BP, MOL and FM groups (*P* < 0.05). *Escherichia*–*Shigella* and *Buchnera* in BP were significant higher than those in NC (*P* < 0.05). *Prevotella_9* in NC were significant higher than those in FM. Meanwhile, *Cetobacterium* in Ref and FM were significantly higher than those in W (*P* < 0.05).

### Clustering dissimilarities

**Fig. 4:**
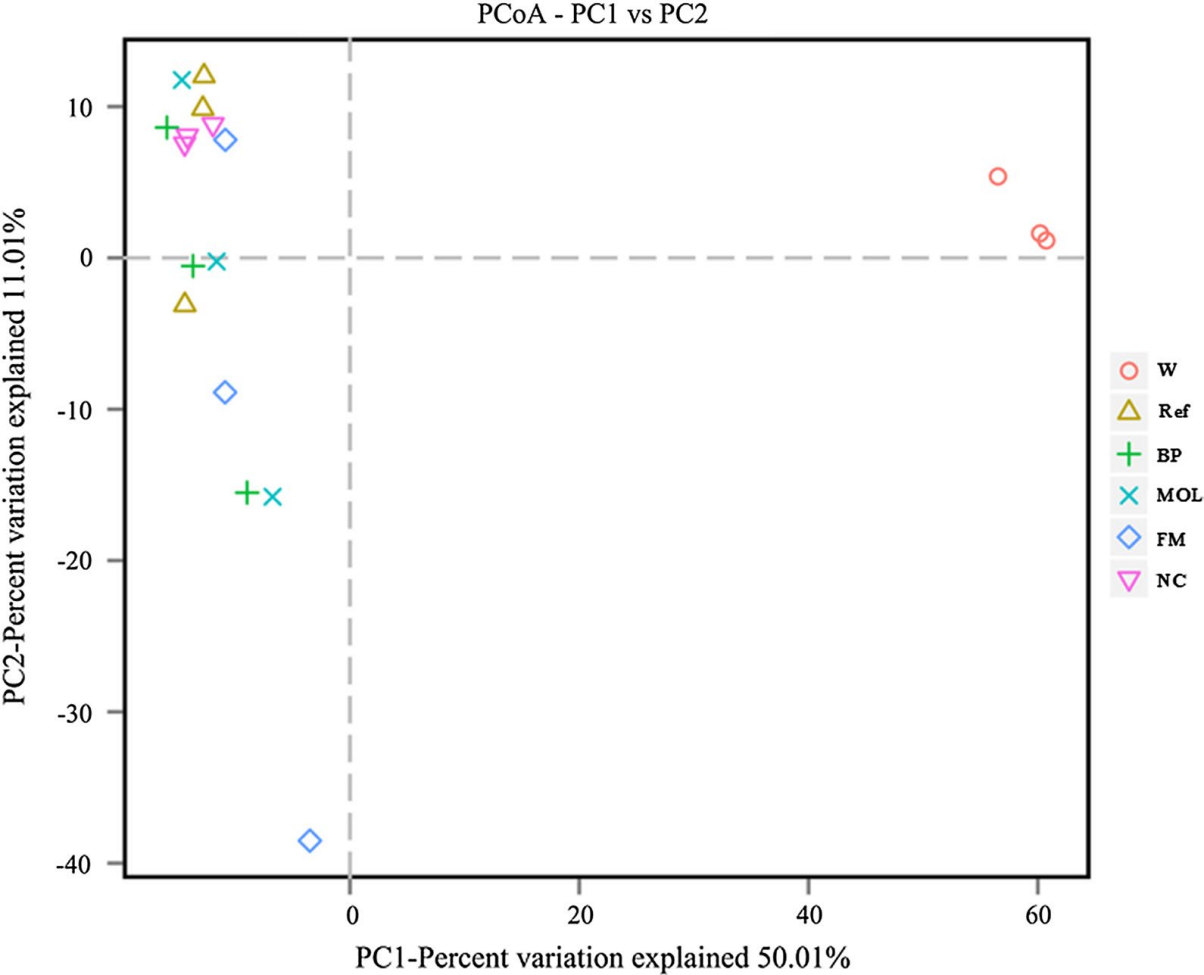
PCoA analyzed the bacteria community. The colored circles represented the bacteria from W, Ref, BP, NC, MOL and FM. PCoA analyzed by with binary Jaccard distances

**Fig. 5:**
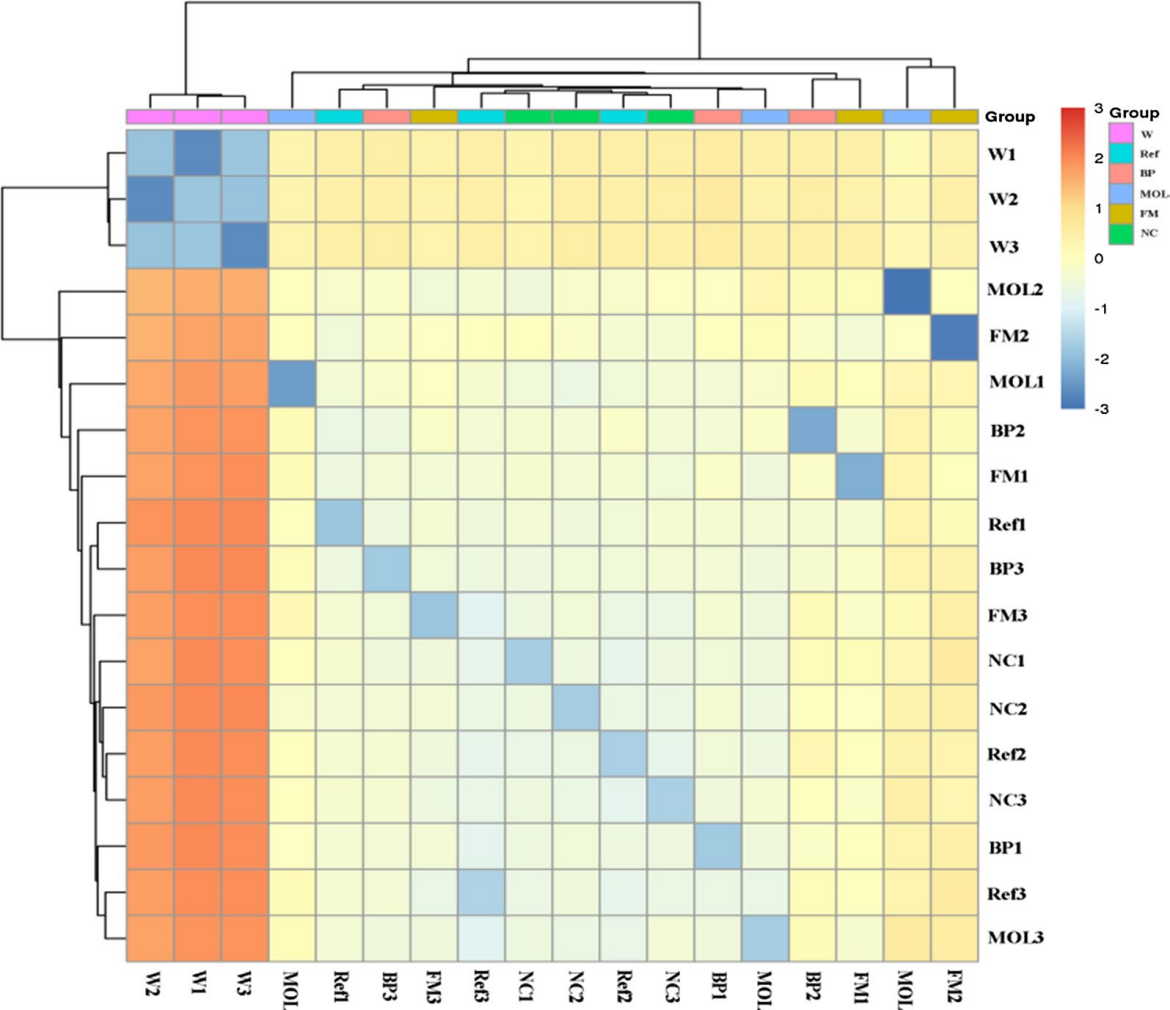
The heatmap of all samples. The different color intensities represented the relative bacteria abundance in W, Ref, BP, NC, MOL and FM groups. Figure was constructed using binary Jaccard distances. The distance of samples gradually increased with the color from blue to red

The principal coordinates analysis (PCoA) indicated that the bacteria community in Ref, BP, NC, MOL and FM clustered together and the bacterial community in Ref, BP, NC, MOL and FM was vaster difference with that in W (Fig. 4). In addition, the heatmap using binary Jaccard distances also showed the similar trend with that of PCoA (Fig. 5). These result showed that the intestinal bacteria community of golden pompano fed with woody forages and reference diet presented higher similarity. The bacteria community in golden pompano intestine was vaster difference from that in W.

### Predicted metabolic pathways and functions

Phylogenetic Investigation of Communities by Reconstruction of Unobserved States (PICRUSt) was used to predict the intestine bacterial functional composition of golden pompano fed with woody forages and reference diet. A total of 338 KEGG orthology (KO) pathways were detected in FM, MOL, NC, BP and Ref groups. Among the 338 pathways, 148, 31, 20 and 18 KEGG pathways were related with metabolism, environmental information processing, genetic information processing and cellular process, respectively. Further analysis the 20 most abundant pathways, four pathways (“arginine and proline metabolism”, “glycine, serine and threonine metabolism”, “valine, leucine and isoleucine degradation”, “alanine, aspartate and glutamate metabolism”) were related with the amino acid metabolism and the glycine, serine and threonine metabolism in FM were significantly higher than those in the Ref, BP, NC and MOL (*P* < 0.05). Six pathways (“butanoate metabolism”, “pyruvate metabolism”, “propanoate metabolism”, “glyoxylate and dicarboxylate metabolism”, “glycolysis/gluconeogenesis”, “carbon metabolism”) were related with carbon metabolism. Two pathways (“purine metabolism”, “pyrimidine metabolism”) were related with nucleotide metabolism. Meanwhile, the two-component system related with signal transduction in BP was significantly higher than those in NC and MOL (*P* < 0.05). ABC transporters related with membrane transport in BP was significantly higher than that in Ref and FM (*P* < 0.05). Oxidative phosphorylation related with energy metabolism in FM was significantly higher than those in NC (*P* < 0.05) (Additional file 1: Table S1).

## Discussion

Intestinal bacteria were involved indigestion, immunity and physiology (Huyben et al. 2017). This study provided the first characterization of intestinal bacteria diversity of golden pompano with illumina-based high-throughput sequencing. And the result indicated that the most phyla of golden pompano intestinal bacteria were *Proteobacteria*, *Firmicutes*, *Fusobacteria* and *Bacteroidetes*. The result was similar with the studies in rainbow trout (Lyons et al. 2015), Eastern African cichlid (Baldo et al. 2015) and Asian carp (Ye et al. 2014). Meanwhile, at the present study, bacterial diversity presented vastly difference between golden pompano intestine and water sample. The result was similar with Johnson et al. (2008) and the phenomenon may be related with the host intestinal environment exerts selective pressure on intestine microbial community establishment (Rungrassamee et al. 2014). The relationship between intestinal bacteria and habitat environmental bacteria was needed further research.

To further analyses the intestinal bacteria dynamic, the abundance of intestinal bacteria of golden pompano fed with woody forages and reference diet were further analyzed. At the present study, the *Proteobacteria* and *Firmicutes* were the dominate phylum. *Proteobacteria* in *Broussonetia papyrifera* group was significantly higher than those in *Neolamarckia cadamba* group and *Firmicutes* in *Neolamarckia cadamba* group were higher than those in *Folium mori* group and *Broussonetia papyrifera* group. The result indicated that the *Broussonetia papyrifera* may be benefit for the *Proteobacteria* and *Neolamarckia cadamba* may be benefit for the *Firmicutes* in golden pompano intestine. Meanwhile, the study indicated that *Proteobacteria* could catabolize feedstuff components (Jumpertz et al. 2011). And the *Firmicutes* were the dominate phylum in the intestine of Eastern African cichlid (Baldo et al. 2015) and Nile tilapia (Jumpertz et al. 2011). The *Bacteroidetes* and *Fusobacteria* presented no significant difference in the woody forages groups and reference groups. Studies indicated that *Bacteroidetes* is and *Fusobacteria* were related with the nutrition metabolism and absorb (Spence et al. 2006). Meanwhile, *Fusobacteria* was found in the intestine of Easter African cichild (Baldo et al. 2015) and grass carp (Ni et al. 2014).

At genera level, *Lactobacillus* was the dominate bacteria of *Firmicutes*. At the present study, the *Lactobacillus* in *Neolamarckia cadamba* group was significantly higher than those in *Folium mori* group and *Broussonetia papyrifera* group. Research indicated that *Lactobacillus* could produce lactic acid which benefit to the health of intestinal tract (Corsetti et al. 1998). *Cetobacterium* were the dominate genera of *Fusobacteria* and study showed that *Cetobacterium* could be helpful for protein digestion and vitamin B12 produce (Finegold et al. 2003). Meanwhile, *Cetobacterium* was also been found in the intestine of grass carp (Hao et al. 2017). *Escherichia*–*Shigella* and *Buchnera* were the dominate genera of *Proteobacteria*. *Prevotella_9* were the dominate genera of *Bacteroidetes*. Studies indicated that *Escherichia*–*Shigella*, *Buchnera* and *Prevotella_9* were belonged to conditional pathogen (Peleg et al. 2008; Hamilton et al. 2013; Scher et al. 2013). These conditional pathogens were also found in yellow catfish and grass carp (Wu et al. 2010; Hao et al. 2017). Meanwhile, *Escherichia*–*Shigella* and *Buchnera* in *Broussonetia papyrifera* group were significant higher than those in *Neolamarckia cadamba* group. And the *Prevotella_9* in *Neolamarckia cadamba* group were significant higher than those in *Folium mori* groups. The phenomenon indicated that the woody forages influence the intestine bacteria abundance and the intestine bacteria were related with the nutrition metabolism and immunity of golden pompano. The further study would be focus on the contribution of intestine bacteria to the physiology and immunity of host.

To further analysis the intestinal bacteria community of golden pompano, the PCoA and heatmap analyses indicated that the woody forages produced less influence on the intestinal bacteria community in this study. However, previous studies indicated that the diet composition could influence the intestinal bacteria community (Schmidt et al. 2016; Yu et al. 2014). The phenomena may because the diets component played the key role in regulating intestinal bacteria community (Huyben et al. 2017).

To further understand the intestinal bacteria metabolism diversity, the intestinal bacteria metabolic function of golden pompano fed with woody forages and reference diet were analyzed and result indicated that biosynthesis of amino acids and carbon metabolism were the dominate intestinal bacteria metabolism pathways of golden pompano. Meanwhile, Ni et al. (2014) also found that the carbon metabolism were also the dominant metabolism pathways of the grass carp intestinal bacteria. ABC transporters in *Broussonetia papyrifera* group were significantly higher than those in the groups of reference and *Folium mori* and study showed that ABC transporters were benefit for the uptake of nutrition (Yan et al. 2016). Meanwhile, in the present study, glycine, serine and threonine metabolism pathways in *Folium mori* group were significantly higher than those in the groups of reference, *Broussonetia papyrifera*, *Neolamarckia cadamba* and *Moringa oleifera* Lam (*P* < 0.05). Meanwhile, amino acid metabolism were also found in the intestinal bacteria of turbot (Xing et al. 2013) and grass carp (Ni et al. 2014). And study showed that the amino acid metabolism of the intestinal bacteria were linearly increased with dietary nutrition levels (Wang et al. 2017). These results indicated that the woody forages influenced on the golden pompano intestinal bacteria metabolism functions and the intestinal bacteria of golden pompano may take participate in the nutrition metabolism. The further relationship between intestinal bacteria and diet digestion and absorb was needed further research.

Therefore, the predominate composition of golden pompano intestine bacteria were *Proteobacteria*, *Firmicutes*, *Fusobacteria* and *Bacteroidetes*. The 30% leafmeals of *Moringa oleifera* Lam, *Broussonetia papyrifera*, *Folium mori*, *Neolamarckia cadamba* influenced the abundance of golden pompano intestinal bacteria in this study. The dominate intestinal bacteria metabolism pathways of golden pompano fed with woody forages and reference diet were biosynthesis of amino acids and carbon metabolism. Meanwhile, the *Broussonetia papyrifera* may be benefit for the nutrition uptake with the ABC transporters metabolism pathway and the *Folium mori* may improve the amino acid metabolism with glycine, serine and threonine metabolism pathway in the intestinal bacteria of golden pompano.

In summary, we first successfully characterized the intestinal bacteria diversity of golden pompano using illumina-based high-throughput sequencing. The further study would be focus on the contributions of intestine bacteria to the physiology and immunity of host. These conclusions are great importance to understand the golden pompano intestinal bacteria diversity and exploit new diets source in aquaculture.

## Additional file

**Additional file 1.** Additional table.

## Abbreviations

MOL: *Moringa oleifera* Lam leaves
BP: *Broussonetia papyrifera* leaves
FM: *Folium mori* leaves
NC: *Neolamarckia cadamba* leaves
Ref: reference diet
CP: crude protein
EE: crude lipid
W: water
QIIME: quantitative insights into microbial ecology
OUTs: operational taxonomic units
RDP: Ribosomal Database Project
ACE: abundance-based coverage estimator
PCoA: principal coordinate analysis
PICRUSt: phylogenetic investigation of communities by reconstruction of unobserved states
SRA: Sequence Read Archive
KO: KEGG orthology
