# Supplementary material for: Woody forages effect the intestinal bacteria diversity of golden pompano Trachinotus ovatus
Source: AMB Express. 2018 Feb 27;8:29. doi: 10.1186/s13568-018-0550-2 (PMC5826908; doi:10.1186/s13568-018-0550-2)
Supplement: Supplementary file 1 — Additional file 1. Additional table. [file 13568_2018_550_MOESM1_ESM.docx]

Table S 1 The 20 most abundant KEGG pathways in MOL, FM, NC, BP and Ref

| KO＃ | KO pathway | Groups | | | | |
| --- | --- | --- | --- | --- | --- | --- |
|  |  | Ref (%) | BP (%) | NC (%) | MOL (%) | FM (%) |
| [ko01230](http://www.kegg.jp/kegg-bin/show_pathway?ko01230) | Biosynthesis of amino acids | 5.8±0.3 | 5.5±0.3 | 5.±0.1 | 5.5±0.3 | 5.7±0.3 |
| [ko01200](http://www.kegg.jp/kegg-bin/show_pathway?ko01200) | Carbon metabolism | 4.4±0.1 | 4.3±0.0 | 4.4±0.0 | 4.4±0.0 | 4.4±0.1 |
| [ko02020](http://www.kegg.jp/kegg-bin/show_pathway?ko02020) | Two-component system | 2.5±0.1^ab^ | 3.0±0.1^a^ | 2.3±0.2^b^ | 2.3±0.2^b^ | 2.6±0.1^ab^ |
| [ko02010](http://www.kegg.jp/kegg-bin/show_pathway?ko02010) | ABC transporters | 3.7±0.1^b^ | 4.0±0.1^a^ | 3.8±0.0^ab^ | 3.8±0.1^ab^ | 3.6±0.1^b^ |
| [ko00230](http://www.kegg.jp/kegg-bin/show_pathway?ko00230) | Purine metabolism | 3.2±0.2 | 2.9±0.1 | 3.6±0.2 | 3.5±0.32 | 3.0±0.04 |
| [ko03010](http://www.kegg.jp/kegg-bin/show_pathway?ko03010) | Ribosome | 3.3±0.2^ab^ | 2.8±0.2^b^ | 3.7±0.3^a^ | 3.7±0.4^a^ | 3.1±0.0^ab^ |
| [ko00330](http://www.kegg.jp/kegg-bin/show_pathway?ko00330) | Arginine and proline metabolism | 1.3±0.1 | 1.5±0.01 | 1.1±0.1 | 1.2±0.2 | 1.5±0.0 |
| [ko00240](http://www.kegg.jp/kegg-bin/show_pathway?ko00240) | Pyrimidine metabolism | 2.5±0.2^ab^ | 2.2±0.1^b^ | 2.9±0.3^a^ | 2.8±0.3^ab^ | 2.3±0.1^ab^ |
| [ko00650](http://www.kegg.jp/kegg-bin/show_pathway?ko00650) | Butanoate metabolism | 1.2±0.1 | 1.3±0.1 | 1.1±0.1 | 1.1±0.1 | 1.3±0.0 |
| [ko01212](http://www.kegg.jp/kegg-bin/show_pathway?ko01212) | Fatty acid metabolism | 1.2±0.1 | 1.3±0.1 | 1.2±0.1 | 1.2±0.0 | 1.2±0.1 |
| [ko00260](http://www.kegg.jp/kegg-bin/show_pathway?ko00260) | Glycine, serine and threonine metabolism | 1.4±0.0^b^ | 1.4±0.0^b^ | 1.4±0.0^b^ | 1.4±0.0^b^ | 1.5±0.1^a^ |
| [ko00280](http://www.kegg.jp/kegg-bin/show_pathway?ko00280) | Valine, leucine and isoleucine degradation | 0.8±0.1 | 1.0±0.1 | 0.7±0.2 | 0.7±0.2 | 1.0±0.1 |
| [ko01210](http://www.kegg.jp/kegg-bin/show_pathway?ko01210) | 2-Oxocarboxylic acid metabolism | 1.3±0.1 | 1.3±0.1 | 1.0±0.1 | 1.1±0.2 | 1.4±0.1 |
| [ko00190](http://www.kegg.jp/kegg-bin/show_pathway?ko00190) | Oxidative phosphorylation | 1.7±0.1^ab^ | 1.7±0.0^ab^ | 1.5±0.1^b^ | 1.6±0.2^ab^ | 1.9±0.1^a^ |
| [ko00620](http://www.kegg.jp/kegg-bin/show_pathway?ko00620) | Pyruvate metabolism | 1.8±0.1 | 1.7±0.01 | 1.9±0.1 | 1.8±0.1 | 1.7±0.1 |
| [ko00640](http://www.kegg.jp/kegg-bin/show_pathway?ko00640) | Propanoate metabolism | 1.2±0.0 | 1.3±0.1 | 1.2±0.0 | 1.2±0.0 | 1.2±0.0 |
| [ko00250](http://www.kegg.jp/kegg-bin/show_pathway?ko00250) | Alanine, aspartate and glutamate metabolism | 1.4±0.1 | 1.3±0.0 | 1.4±0.1 | 1.4±0.1 | 1.3±0.1 |
| [ko00630](http://www.kegg.jp/kegg-bin/show_pathway?ko00630) | Glyoxylate and dicarboxylate metabolism | 1.1±0.1 | 1.2±0.1 | 0.9±0.1 | 1.0±0.1 | 1.2±0.1 |
| [ko00010](http://www.kegg.jp/kegg-bin/show_pathway?ko00010) | Glycolysis / Gluconeogenesis | 1.7±0.1 | 1.5±0.0 | 1.9±0.1 | 1.8±0.2 | 1.5±0.1 |
| [ko02040](http://www.kegg.jp/kegg-bin/show_pathway?ko02040) | Flagellar assembly | 0.7±0.2 | 0.9±0.1 | 0.5±0.2 | 0.6±0.2 | 0.8±0.1 |

In the same row, values with different letter superscripts mean significant differences (*P*<0.05).
